# Rifampicin Enhanced Carbapenem Activity with Improved Antibacterial Effects and Eradicates Established *Acinetobacter baumannii* Biofilms

**DOI:** 10.3390/ph16040477

**Published:** 2023-03-23

**Authors:** Lois Chinwe Nwabor, Arnon Chukamnerd, Ozioma Forstinus Nwabor, Rattanaruji Pomwised, Supayang P. Voravuthikunchai, Sarunyou Chusri

**Affiliations:** 1 Department of Biomedical Sciences and Biomedical Engineering, Faculty of Medicine, Prince of Songkla University, Songkhla 90110, Thailand; 2 Division of Infectious Diseases, Department of Internal Medicine, Faculty of Medicine, Prince of Songkla University, Songkhla 90110, Thailand; 3 Division of Biological Science, Faculty of Science, Prince of Songkla University, Songkhla 90110, Thailand; 4 Natural Product Research Center of Excellence, Faculty of Science, Prince of Songkla University, Songkhla 90110, Thailand; 5 Center of Antimicrobial Biomaterial Innovation—Southeast Asia, Prince of Songkla University, Songkhla 90110, Thailand

**Keywords:** rifampicin-resistant and carbapenem-resistant *Acinetobacter baumannii*, combination therapy, antibiofilm, antibacterial

## Abstract

Biofilm-mediated infections are critical to public health and a leading cause of resistance among pathogens, amounting to a prolonged hospital stay and increased mortality rate in the intensive care unit. In this study, the antibacterial and antibiofilm activities of rifampicin or carbapenem monotherapies were compared with rifampicin and carbapenem combination therapies against rifampicin-resistant and carbapenem-resistant *Acinetobacter baumannii* isolates. Among 29 CRAB isolates, 24/29 (83%) were resistant to rifampicin, with MIC values between 2–256 µg/mL. Checkerboard assays disclosed that combination therapies at FICIs between 1/8 and 1/4 improved the activity of carbapenems at subinhibitory concentrations. Time-kill kinetics indicated a 2- to 4-log reduction at 1/2 MIC rifampicin + 1/4 MIC carbapenem and 1/4 MIC rifampicin + 1/4 MIC carbapenem against the isolates, with the MIC values ranging from 2–8 µg/mL. The MTT assay revealed a dose-dependent decrease of the cell viability of established bacterial biofilm at 4 MIC rifampicin + 2 MIC carbapenems, with a percentage reduction of 44–75%, compared with monotherapies at 16 MIC. Scanning electron microscopy further confirmed bacterial cell membrane disruption, suggesting a synergism between carbapenem and rifampicin against a representative isolate. The findings demonstrated that the combination of rifampicin with carbapenems could improve antibacterial activities and eradicate established *Acinetobacter baumannii* biofilm.

## 1. Introduction

In the pharmaceutical industry, biofilm formation has become a concern and one of the major causes of antimicrobial resistance among clinically relevant pathogens, such as *Acinetobacter baumannii*. Carbapenem-resistant *Acinetobacter baumannii* (CRAB) has emerged as a global health challenge that threatens human health with an increasing death toll and healthcare costs [1]. Current estimates suggest a 97.6% occurrence of extensive drug resistance among *A. baumannii* clinical isolates, with 2.4% demonstrating pan-drug resistance [2], whereas a more recent study revealed a 100% occurrence of extensive drug resistance among isolates [3]. The ability of CRAB to acquire antimicrobial resistance genes and upregulate virulence-associated genes aggravates the antimicrobial-resistant threat, with several cases of treatment failure [4]. Most *A. baumannii* isolates obtained from clinical specimens, such as sputum, urine catheters, cerebrospinal fluid (CSF), bronchoalveolar lavage exudates, and burned skin, recently exhibited increased biofilm formation ability [5]. Infected indwelling medical devices, such as catheters, endotracheal tubes, oropharynx, tracheostomy, cardiac valves, artificial joints, and ventilators, are prone to microbial colonization and biofilm formation, which is responsible for biofilm-associated complications, such as ventilator-acquired pneumonia, meningitis, bacteremia, and catheter-associated urinary tract infection [5,6]. The infectivity of biofilm-forming pathogens accounts for about 65–80% of the human infection rate [7]. Patients in the intensive care unit (ICU) are prone to reinfection by biofilm-associated pathogens, which are difficult to eradicate as a result of desiccator and disinfectant resistance. In addition, several researchers have revealed the role of biofilm formation in CRAB, and recently, *A. baumannii* was described among the most prevalent organisms associated with biofilm on tracheostomy tubes in the ICU [6,8]. The survival of *A. baumannii* in both natural environments and artificial surfaces has been linked to its ability to form a resistant biofilm, which is often promoted by cell-to-cell interaction, and quorum sensing [9,10]. The biofilm environment protects organisms against antibiotics, bacteriophage, and other antimicrobial agents. Resistance to antibiotic treatment by sessile cells has been linked with alterations in cells [11]. Biofilm-mediated resistance was recently detected among colistin-resistant and carbapenem-resistant *A. baumannii* isolates [12]. Established or matured biofilms are more resistant to antibacterial agents as more microbial aggregation occurs, leading to the overexpression of the extracellular polymeric substances (EPSs), a biofilm-specific efflux pump, and reduced antibiotic diffusion [13]. These factors contribute to the increased biofilm eradication concentration. A recent study reported a 32- to 256-fold increase between MBC of planktonic cells and MBIC of biofilm cells [14]. In medical practice, carbapenems are used as a last-resort antibiotic for the management of *A. baumannii* infections. Carbapenems are treatment options due to their broad spectrum of activity and associated stability to hydrolytic enzymes. Carbapenems prevent peptide cross-linking and other peptidase events by blocking the peptidase domain of penicillin-binding proteins during cell wall synthesis. Recent data from the national antimicrobial resistance surveillance report in Thailand estimated over 70% resistance to imipenem in Asia, indicating that carbapenems may become ineffective for the treatment of *Acinetobacter* spp. However, strategizing treatment measures aimed at managing antimicrobial-resistant bacterial infection employ antimicrobial repurposing and combination therapy to mitigate mortality and other clinical outcomes due to antibiotic resistance. Combination therapy could be a promising treatment option to broaden the antimicrobial spectrum and provides diverse inactivation mechanisms for pathogenic bacteria. Rifampicin is a broad-spectrum and bactericidal antibiotic used for the treatment of *Mycobacterium* spp. and *Hemophilus influenza* and in adjunctive therapy with other antibiotics for the management of *Staphylococcus* spp. and *E. coli* infections [15]. Rifampicin blocks the transcription elongation via cleavage to the beta subunit of the RNA polymerase of prokaryotic cells. Rifampicin has been shown to penetrate biofilm as it circulates throughout the body system, including the CSF [16,17]. It is recommended that it should be used in combination with other antibiotics, based on the high resistance potential (CLSI, 2020). Rifampicin adjunctive therapy has demonstrated synergism with other antimicrobial agents against resistant clinical isolates at subinhibitory concentrations, with an ability to inhibit biofilm formation and development [16,18].

This research is aimed at investigating the antibacterial and antibiofilm ability of rifampicin-based combination therapy with carbapenems against rifampicin-resistant and carbapenem-resistant *A. baumannii* (RR-CRAB) clinical isolates.

## 2. Results and Discussion

### 2.1. Bacteria Isolates

The demographic data of patients infected by carbapenem-resistant *Acinetobacter baumannii* indicate that all the patients have at least one underlying health disease and hospitalization for ≥6 days. The patients were all admitted to the intensive care unit of the hospital with a history of prior antibiotic use, including meropenem, imipenem, erythromycin, piperacillin, levofloxacin, and/or ciprofloxacin.

### 2.2. Antibacterial Effects of Rifampicin and Carbapenems on CRAB Clinical Isolates

The activity of rifampicin on the CRAB clinical isolate was assessed using the disc diffusion test, and the results are presented in Figure S1. The isolates were further tested with a broth microdilution assay. The disc diffusion results showed that 16 of 218 CRAB clinical isolates demonstrated resistance to rifampicin, with no zone of inhibition, while 202 others displayed zones of inhibition between 5 mm to 25 mm. Isolates with zones of inhibition above 18 were dropped from the study. Since there is no EUCAST nor CLSI breakpoint for rifampicin on *A. baumannii*, the breakpoint recorded on *Staphylococcus aureus* was adopted. A total of 89%, 9%, and 2% of the isolates enrolled for the study were resistant, intermediate, and susceptible to rifampicin, based on the disc diffusion assay. Overall, 29 isolates with zones of rifampicin inhibition between 0–18 mm were used for further studies. Previous studies have reported a high resistance of *A. baumannii* isolates from Thailand to various antimicrobial agents, including gentamycin, tobramycin, minocycline, fosfomycin, carbapenems, ciprofloxacin, trimethoprim/sulfamethoxazole, linezolid, and colistin [19,20,21]. In this study, almost all the isolates were resistant to both rifampicin and carbapenems. Resistance to rifampicin has been detected in 84.2% of the multidrug-resistant *A. baumanii* (MDR-AB) isolates [22]. In another study, rifampicin also displayed a MIC range between 4–128 µg/mL against *A. baumannii* isolates, indicating a high level of rifampicin resistance [23]. The MICs of rifampicin, meropenem, imipenem, and doripenem against our CRAB isolates are presented in Table 1. 

The rifampicin MICs of the isolates ranged from 1 to 256 µg/mL, from which 14 out of 29 isolates were inhibited at concentrations of 1–8 µg/mL of rifampicin, while 15 isolates were inhibited at MICs of 16–256 µg/mL of rifampicin. In addition, all the isolates were resistant to meropenem, doripenem, and imipenem at MICs of 16–128 µg/mL, 8–128 µg/mL, and 16–512 µg/mL, respectively. The standard strain, ATCC 19606, demonstrated susceptibility to rifampicin and carbapenems at a MIC of <2 µg/mL. Finally, 24 isolates were classified as rifampicin-non-susceptible CRAB isolates, and the results indicated resistance to antibiotics with diverse modes of action, including cell wall inhibition, and RNA polymerase inhibition.

### 2.3. Checkerboard Assay

The efficacy of rifampicin in combination with meropenem, doripenem, and imipenem was assessed in all 29 isolates of rifampicin-resistant CRAB. The factional inhibitory concentration index (FICI) of a few isolates showed synergism, while most isolates were additive and indifferent to treatment (Table 1). The antibacterial interaction between meropenem and rifampicin showed synergism in about 7/29 of the isolates, 21/29 were additive, and 1/29 was indifferent. Imipenem and rifampicin combination indicated an improved activity compared to other combinations, with 10/29 synergism, 16/29 additive, and 3/29 indifferent against CRAB isolates. For the doripenem and rifampicin combination, 24/28 of the isolates were additive, while 2/28 of the isolates were synergistic and indifferent, respectively. Most of the isolates with rifampicin MICs of >8 µg/mL were not susceptible to the combinations of rifampicin with carbapenems, while isolates with rifampicin MIC of ≤8 µg/mL responded with a four- to eightfold reduction. The result suggested that rifampicin–carbapenem combinations are more effective against CRAB isolates with lower rifampicin MICs. This corresponds to previous work on rifampicin in combination with cefoperazone-sulbactam, an antibiotic of the same class as carbapenem [22]. The combination of rifampicin with imipenem or doripenem against TR123 was synergistic, indicating that rifampicin enhanced the activity of the antibiotics in combination. However, the concentrations of imipenem plus rifampicin were still higher than the breakpoint from CLSI (2020). Although rifampicin is currently not indicated for the treatment of *A. baumannii* infections, studies have shown that combinations of rifampicin promote the antibacterial potency of other antibiotics [16,24]. However, it has been recommended that rifampicin should not be used alone to avoid the development of resistance in most pathogens of clinical relevance (CLSI, 2020). In addition, rifampicin is a biofilm active agent, which inhibits transcription at the β-subunit of RNA-polymerase and imposes bactericidal activity on diverse bacteria species, while carbapenems inhibit the cross-linking of penicillin-binding proteins (PBPs), halting de novo synthesis of peptidoglycan, and have a high permeabilizing effect.

### 2.4. Time-Kill Assay

**Figure 1:**
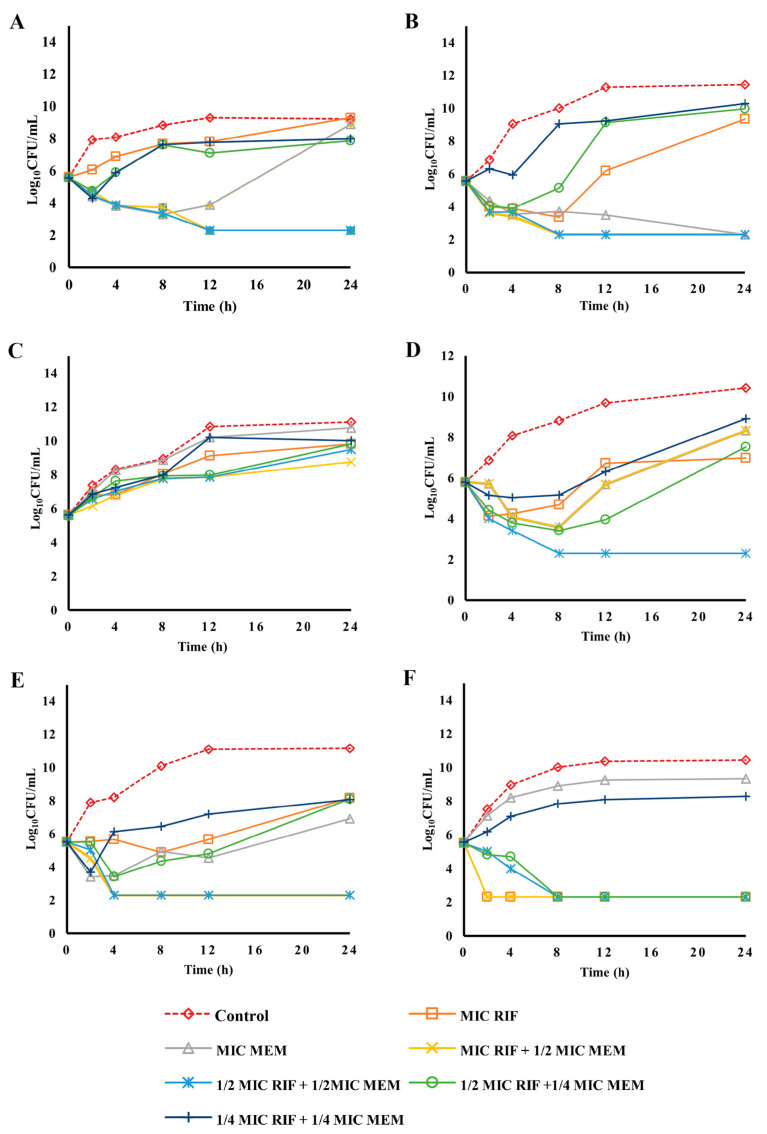
Time-kill kinetic of rifampicin and combination with meropenem against rifampicin-resistant and carbapenem-resistant clinical isolates of *A. baumannii*. SK015 (**A**), TR069 (**B**), and ST004 (**C**) are isolates with high rifampicin MIC (≥16 µg/mL), while TR131 (**D**), TR123 I (**E**), and TR057 (**F**) are isolates with lower rifampicin MIC (≤8 µg/mL). Experiments were performed in duplicate. MIC, minimum inhibitory concentration; RIF, rifampicin; MEM, meropenem.

The time-kill assay was conducted to investigate the killing dynamics of the antibiotic combinations. The results revealed both bacteriostatic and bactericidal effects of combinations of carbapenems with rifampicin. In total, 7 isolates used in the time-kill assay were selected from isolates with high (≥32 µg/mL) and lower (2–8 µg/mL) rifampicin MICs. In Figure 1A,B,D, rifampicin and meropenem combination resulted in bactericidal effects at MIC rifampicin + 1/2 MIC meropenem and 1/2 MIC rifampicin + 1/2 MIC meropenem, with a greater than 4-log reduction and 3-log reduction, respectively, for SK015, TR069, and TR131. Combinations of 1/2 MIC rifampicin and 1/4 MIC meropenem were bacteriostatic against TR131 but showed no effects against SK015 and TR069 after 12 h of incubation. Meropenem alone at MIC exhibited >99% (2.5 log) killing against SK015. This report agrees with a previous study with meropenem alone exhibiting a 40% and rifampicin alone exhibiting a 10% bactericidal effect at MIC on CRAB isolates [25]. Combination of meropenem and rifampicin against SK024 resulted in synergistic effects at 1/4 MIC rifampicin and 1/4 MIC meropenem after 8 h (Figure S2). However, a regrowth was observed at 12 h, indicating a bacteriostatic effect. The MICs of individual antibiotics and combinations of subinhibitory concentrations were ineffective against ST004 (Figure 1C). Rifampicin and meropenem combinations at MIC rifampicin + ½ MIC meropenem and ½ MIC rifampicin + ½ MIC meropenem were bactericidal against TR123 and TR057 with a 4-log reduction (Figure 1E,F). Although a bactericidal effect was observed at the resistant breakpoint of rifampicin, combination therapy at ½ MIC rifampicin + ¼ MIC meropenem against the studied isolates is preferable since rifampicin single therapy is discouraged. Combination of ½ MIC rifampicin + ½ MIC meropenem was also bactericidal in the same referenced study [25].

**Figure 2:**
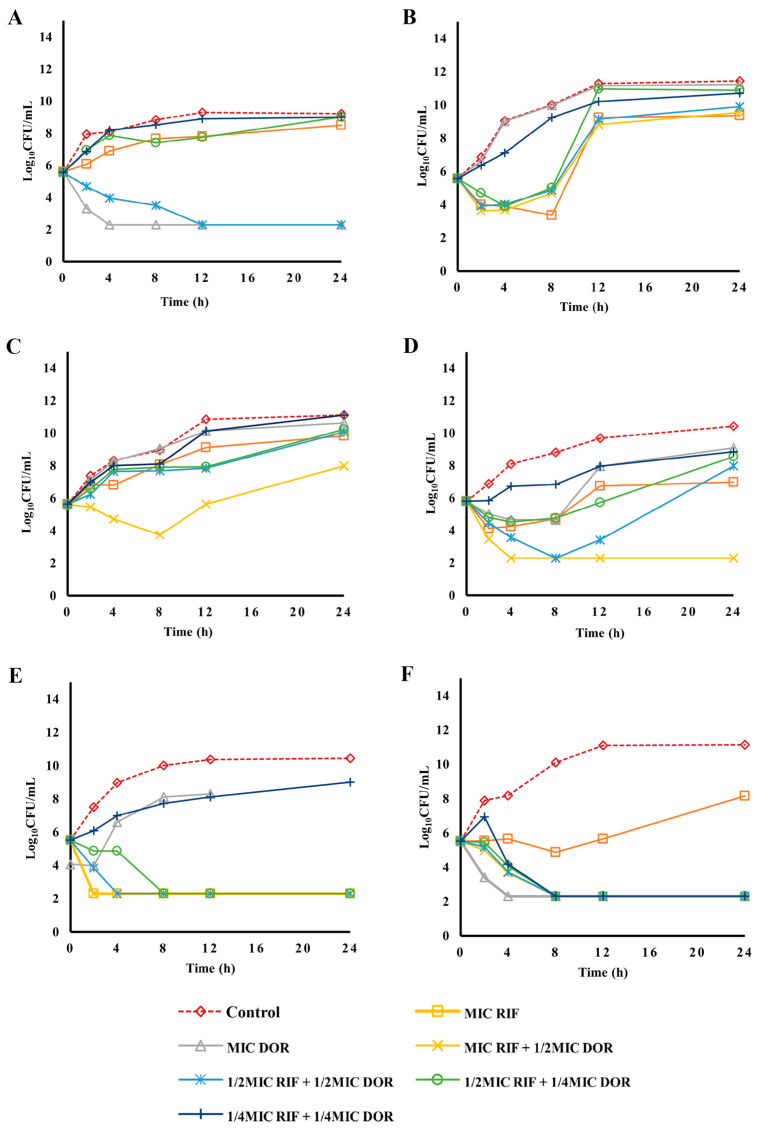
Time-kill kinetics of rifampicin and combination with doripenem against rifampicin-resistant and carbapenem-resistant clinical isolates of *A. baumannii*. SK015 (**A**), TR069 (**B**), and ST004 (**C**) are isolates with high rifampicin MIC (≥16 µg/mL), while TR131 (**D**), TR123 (**E**), and TR057 (**F**) are isolates with lower rifampicin MIC (≤8 µg/mL). Experiments were performed in duplicate. MIC, minimum inhibitory concentration; RIF, rifampicin; DOR, doripenem.

The result of rifampicin in combination with doripenem was less potent against isolates with a high rifampicin MIC (≥32 µg/mL), compared to other carbapenems (Figure 2A–C). Among isolates with lower rifampicin MICs, a bactericidal effect of MIC rifampicin + 1/2 MIC meropenem against SK015 only was observed. At MIC rifampicin + 1/2 MIC meropenem and 1/2 MIC rifampicin + 1/2 MIC meropenem, a bactericidal effect was observed after 12 h of incubation for TR131. The effect of 1/2 MIC rifampicin + 1/4 MIC meropenem demonstrated an early effect at 8 h, but a regrowth was observed at 12 h (Figure 2D). Similarly, 1/4 MIC rifampicin + 1/4 MIC meropenem, rifampicin MIC, and doripenem MIC were ineffective against TR131. Treatments of TR123 and TR057 at MIC rifampicin + 1/2 MIC meropenem, 1/2 MIC rifampicin + 1/2 MIC meropenem, 1/2 MIC rifampicin + 1/4 MIC meropenem, and 1/4 MIC rifampicin + 1/4 MIC meropenem resulted in a bactericidal effect, with a 4-log reduction (Figure 2E,F). Furthermore, synergistic activity was observed for combination at 1/4 MIC rifampicin + 1/4 MIC doripenem against TR123, resulting in a bactericidal effect (Figure 2E).

**Figure 3:**
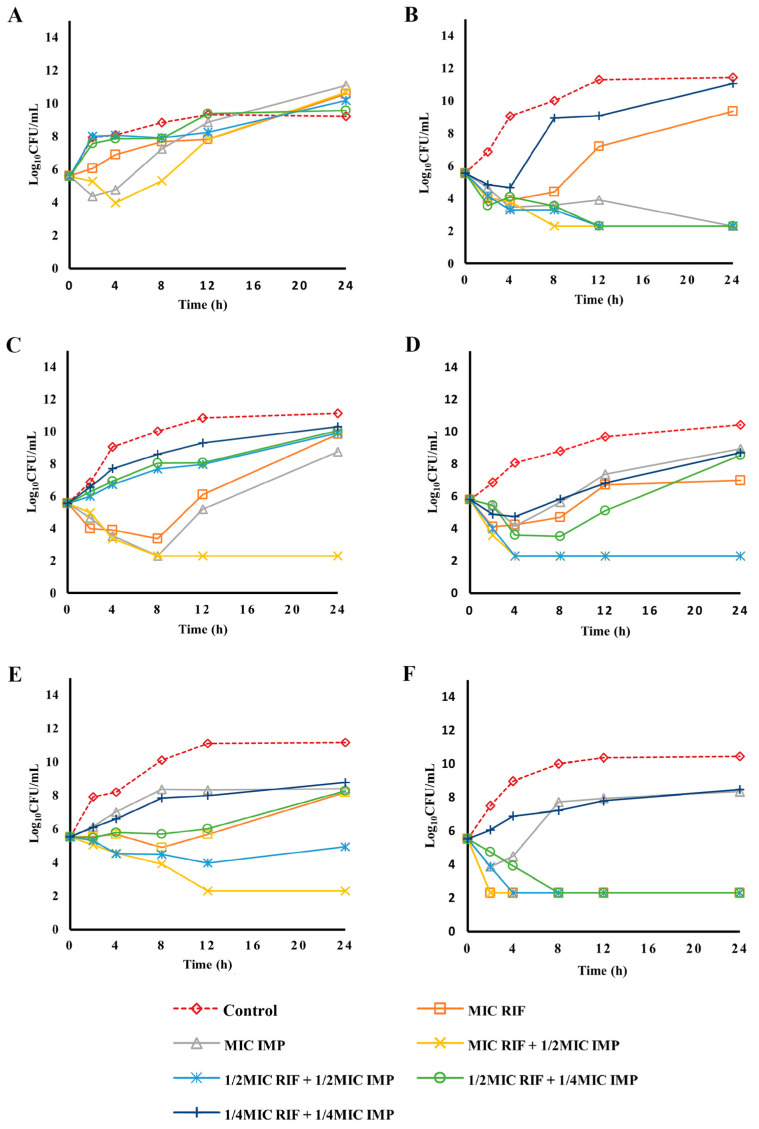
Time-kill kinetics of rifampicin and combination with imipenem against rifampicin-resistant and carbapenem-resistant clinical isolates of *A. baumannii*. SK015 (**A**), TR069 (**B**), and ST004 (**C**) are isolates with high rifampicin MIC (≥16 µg/mL), while TR131 (**D**), TR123 (**E**), and TR057 (**F**) are isolates with lower rifampicin MIC (≤8 µg/mL). Experiments were performed in duplicate. MIC, minimum inhibitory concentration; RIF, rifampicin; IMP, imipenem.

Rifampicin plus imipenem combination showed an indifferent effect against SK015 (Figure 3A), but it was bactericidal, with a fourfold reduction of TR069 at MIC rifampicin + 1/2 MIC imipenem, 1/2 MIC rifampicin + 1/2 MIC imipenem, and 1/2 MIC rifampicin + 1/4 MIC imipenem (Figure 3B). Rifampicin monotherapy was ineffective, whereas imipenem monotherapy resulted in a bactericidal effect, with a 3-log reduction (Figure 3B). MIC rifampicin + 1/2 MIC imipenem and 1/2 MIC rifampicin + 1/2 MIC imipenem demonstrated a bactericidal effect for TR131, whereas combination against ST004 and TR131 was at a subinhibitory concentration (Figure 3C,D). The combination of rifampicin plus imipenem resulted in a bactericidal effect against TR123 at MIC rifampicin plus 1/2 MIC imipenem. The activity of 1/2 MIC rifampicin plus 1/2 MIC imipenem was bacteriostatic. Other combinations were inactive against TR123. (Figure 3E). Almost all concentrations of rifampicin plus imipenem combinations were bactericidal against TR057 except for 1/4 MIC rifampicin + 1/4 MIC imipenem. In addition, rifampicin alone displayed a bactericidal effect against TR057. (Figure 3F). Based on the mechanisms of actions of the antibiotics, carbapenems may have disrupted the bacteria cell membrane, enabling rifampicin accumulation and enhancing antibacterial activity.

### 2.5. Biofilm Inhibitory Activity of Rifampicin with Carbapenems

Biofilm formation was assessed among 29 CRAB clinical isolates. Based on a crystal violet assay, the results indicated that 100% of the isolates were biofilm formers, of which 52% were strong biofilm formers, 34% were moderate biofilm formers, and 14% were weak biofilm formers (Table S1). This result is similar to the finding of Sarshar et al. that 75–100% of *A. baumannii* isolates were biofilm formers [26,27]. 

**Figure 4:**
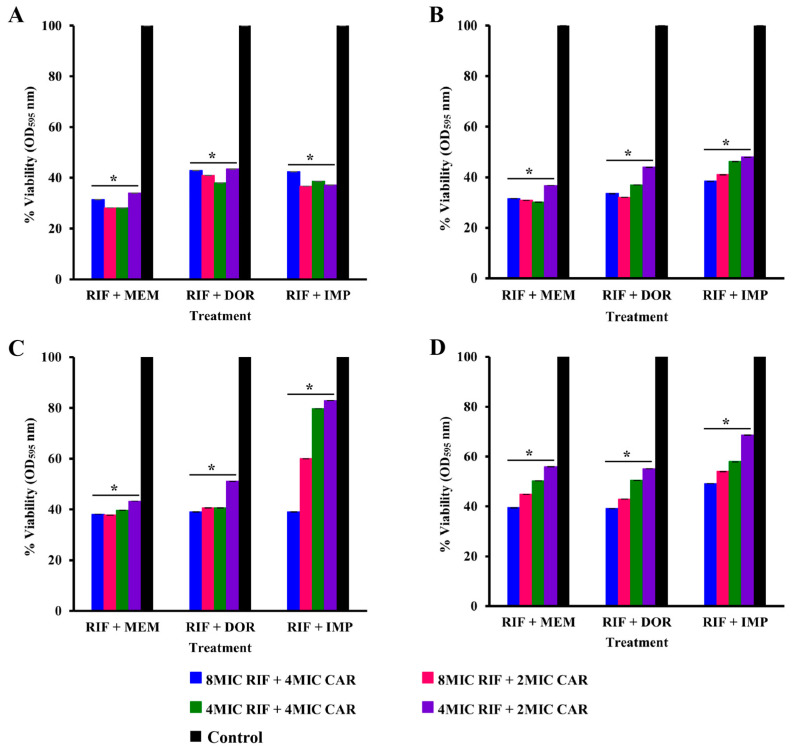
Cell viability of combination therapy against 96 h-established biofilm (**A**) SK015, (**B**) SK024, (**C**) TR125, and (**D**) TR045 expressed as percentage viability. MIC, minimum inhibitory concentration; RIF, rifampicin; MEM, meropenem; DOR, doripenem; IMP, imipenem; CAR, carbapenems. * *p* < 0.05.

The antibiofilm activities of antibiotics and their combinations were studied on the 96 h-established biofilm of 6 strong biofilm-forming isolates, including ATCC 19606. The biofilm disrupting activity was then examined on 96 h-established biofilm, and the viability of biofilm cells was quantified using MTT assays. A concentration-dependent reduction in the number of viable cells was observed for all isolates compared with the untreated control. Treatments with a single antibiotic at 16, 8, and 4 MICs were ineffective against established biofilm compared to antibiotic combinations. Combinations decreased the number of viable cells of 96 h-established biofilm at 8 MIC + 4 MIC, 8 MIC + 2 MIC, 4 MIC + 4 MIC, and 4 MIC + 2 MIC rifampicin plus carbapenems respectively on all isolates (Figure 4 and Figure S3).

**Figure 5:**
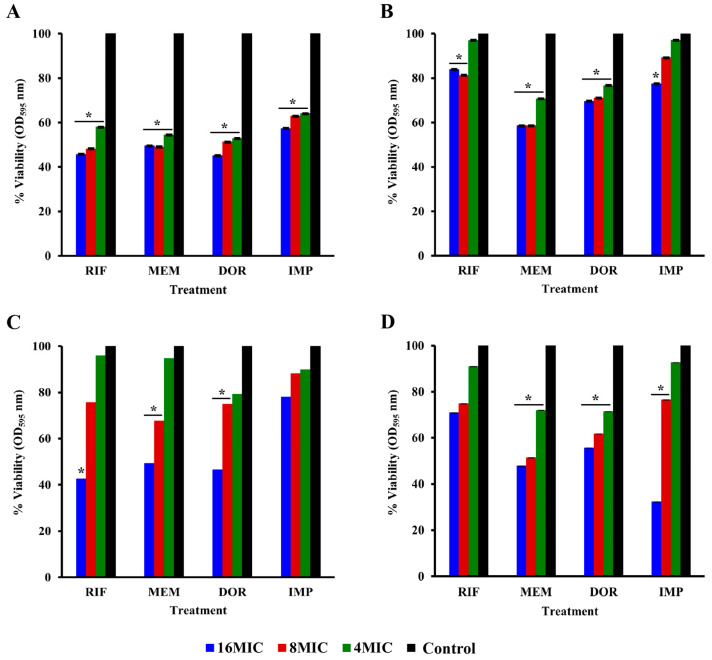
Cell viability of monotherapy against 96 h-established biofilm (**A**) SK015, (**B**) SK024, (**C**) TR125, and (**D**) TR045 expressed as percentage viability. MIC, minimum inhibitory concentration; RIF, rifampicin; MEM, meropenem; DOR, doripenem; IMP, imipenem. * *p* < 0.05.

The MTT assay of rifampicin monotherapies on 96 h-established biofilm revealed that at 16 MIC, the number of viable biofilm cells reduced by 16–57% among the isolates and ATCC 19606. In a previous study, rifampicin demonstrated an enhanced antibiofilm effect against the established biofilm of *A. baumannii* clinical isolates [28]. However, our study indicated that meropenem and doripenem were more effective against *A. baumannii* biofilms (Figure 5A).

This might be because of the lower molecular weights of carbapenems, which enables them to effectively penetrate and accumulate in the cell at a therapeutic concentration. At 16 MIC of meropenem, a 40–52% reduction in viable biofilm cells was observed against the isolates. Doripenem and imipenem single therapy also decreased the viability of biofilm cells in a dose-dependent manner, resulting in 27–70% and 22–68% reduction, respectively. Single antibiotic treatment demonstrated less potency against the tested isolates at a higher concentration, whereas combination at lower concentrations significantly reduced (*p* < 0.05) the viability of biofilm cells (Figure 5 and Figure S3).

In the present study, at 4 MIC rifampicin combined with 2 MIC meropenem, the viabilities of biofilms were reduced by 44–74% compared to single antibiotic therapies of either rifampicin or meropenem at 16 MIC (Figure 5A). Similarly, 4 MIC rifampicin combined with 2 MIC imipenem was also more effective than single antibiotic treatments at 16 MIC, with a 42–72% reduction in biofilm viability. The antibiofilm activities of imipenem and rifampicin have previously been reported against *A. baumanni* [16]. Combination of rifampicin and doripenem exhibited antibiofilm effects against the 96 h-established biofilm, with a 44–75% reduction in the biofilm viability of the tested isolates and standard strain compared to doripenem monotherapy (Figure 4 and Figure S4).

The combinations of carbapenem with rifampicin resulted in promising antibiofilm effects against *A. baumannii* clinical isolates. The cumulative effects of the combinations synergistically facilitated the disruption of the bacterial biofilm. Previously, studies have shown that carbapenem-resistant *A. baumannii* clinical isolates in the biofilm demonstrated resistance, with higher minimum inhibitory concentrations of both single and combined antibiotic treatments compared with cells in suspension [14,29]. In addition, it has been found that the limited antibiotic susceptibility in biofilm environments is related to the selectivity of antibiotics by the bacteria outer membrane structure, decreased antibiotic diffusion due to bacteria aggregation, altered microbial phenotype, and genotypic features during cell-to-cell interactions [30]. Antimicrobial strategies with several targets broaden the antibacterial spectrum, while reducing the resistance window. The use of rifampicin in combination therapies provides therapeutic benefits by reducing its side effects, such as hepatotoxicity, eliminating the emergence of rifampicin resistance, as well as broadening its spectrum of activity. Despite the limited uptake of rifampicin, combination therapies with carbapenem have been shown to decrease the activity of the outer membrane barrier, allowing rifampicin to reach the targeted RNA polymerase.

### 2.6. Scanning Electron Microscopy

**Figure 6:**
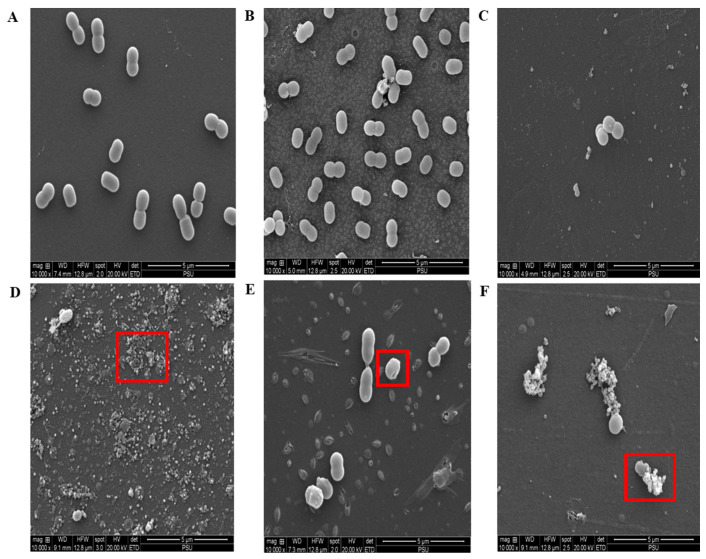
Scanning electron micrograph of the SK024 isolate after 3 h treatment with rifampicin and meropenem alone and in combinations at 10,000 × magnification. (**A**) untreated cells, (**B**) treatment with rifampicin alone, (**C**) treatment with meropenem alone, (**D**) treatment with MIC rifampicin + 1/2 MIC meropenem, (**E**) treatment with 1/2 MIC rifampicin + 1/2 MIC meropenem, and (**F**) treatment with 1/2 MIC rifampicin + 1/4 MIC meropenem.

The activity of antibiotics on the bacteria cell membranes revealed a synergistic disruptive effect of the rifampicin and meropenem combination against SK024 (Figure 6). 

The cell membrane disruption indicates that rifampicin enhances meropenem activity, leading to the breakdown of bacterial membranes and an increased influx of antibiotics. This result is in congruence with a previous report on rifampicin combination with dalbavancin, which is similar to carbapenems, by inhibiting bacterial peptidoglycan synthesis [28]. Combination therapy at MIC rifampicin and 1/2 MIC meropenem destroyed most of the bacteria cell, as shown by the presence of cell debris (Figure 6D). Combination also resulted in the changes in the cell structure leading to the elongation of bacteria cells at 1/2 MIC rifampicin and 1/2 MIC meropenem (Figure 6E) after 3 h of incubation. Compared to the control, there were a limited number of bacteria cells when treated with antibiotics in combinations in a concentration-dependent manner. Further, the combination of 1/2 MIC rifampicin and 1/4 MIC meropenem was bactericidal against SK024 after 3 h of incubation.

## 3. Materials and Methods

### 3.1. Chemicals and Media

Imipenem and meropenem were procured from Siam Bheasach Co., Ltd., (Chomphon) (Chatuchak, Bangkok, Thailand), and doripenem was obtained from Shionogi pharma Co., Ltd. Kanegasaki plant, (Iwate, Japan). Culture media, including Mueller Hinton Agar (MHA), Mueller Hinton Broth (MHB), Tryptic Soy Agar (TSA), and Tryptic Soy Broth (TSB), were supplied by Becton Dickinson & Co. Difco (Franklin Lakes, NJ, USA) USA. Rifampicin was purchased from HiMedia Laboratories Pvt. Ltd., (Mumbai, India).

### 3.2. Bacterial Isolates

Previously characterized *A. baumannii* clinical isolates collected from patients with diverse health comorbidities, such as chronic obstructive pulmonary disease, chronic kidney disease, coronary heart disease, cerebrovascular disease, diabetes mellitus, dyslipidemia, essential hypertension, and pulmonary heart disease, were utilized in the study. Samples were isolated from sputum, skin, wounds, urine, and blood. All isolates were also previously identified as CRAB (n = 219) through a disc diffusion test [20]. Isolates were stored at −80 °C and subcultured in TSA. *Acinetobacter baumannii* ATCC 19606, which is a susceptible and strong biofilm former, was used as a control in the study.

### 3.3. Screening for Rifampicin Resistance in CRAB Isolates

The disc diffusion test was used to identify rifampicin-resistant isolates. A colony of each isolate was inoculated into MHB and incubated for 5 h to log phase. The optical density (OD) was measured, converted to 0.5 MacFarland’s standard, and further adjusted to 10^6^ CFU/mL. Then, a 100 µL aliquot of culture was evenly spread on MHA plates, and a rifampicin disc of 5 µg was placed on each of the plates. The plates were then incubated overnight at 37 °C. Since there is no CLSI breakpoint for rifampicin on *A. baumannii,* isolates with a 0–18 mm zone of inhibition were enrolled in the study [31].

### 3.4. Antibacterial Testing of RR-CRAB Isolates

CRAB isolates that were resistant to rifampicin were used in the study. Isolates were treated with carbapenems (imipenem, meropenem, and doripenem) and rifampicin. A broth micro-dilution assay was employed to assess the minimum inhibitory concentrations (MICs) of the antibiotics on rifampicin-resistant and carbapenem-resistant isolates (RR-CRAB), in accordance with the CLSI guideline (CLSI, 2020). Briefly, 100 µL of 10^6^ CFU/mL bacterial culture was treated with various antibiotic concentrations in a 96-well microtiter plate [32]. For each experiment, both positive and negative controls, consisting of wells with antibiotics alone and bacteria alone, were included. The plates were incubated at 37 °C for 18 h, and bacterial inhibition was assessed using the resazurin test. MIC was expressed as the lowest concentration of the antibiotic that inhibited the bacteria growth.

### 3.5. Antimicrobial Combination Assays

The synergistic activity of rifampicin with imipenem, meropenem, and doripenem on carbapenem-resistant isolates of *A. baumannii* was evaluated using the checkerboard technique. Briefly, bacterial suspensions were treated with 50 µL of rifampicin and 50 µL of either imipenem, doripenem, or meropenem at corresponding concentrations in a 96-well microtiter plate. The plates were then incubated at 37 °C for 18 h. Inhibitory concentrations were determined as concentrations without growth, as indicated by the resazurin test. The antibacterial effects of single antibiotics were also tested as a control. The experiment was performed for three independent repeats. The antimicrobial efficacy of the combination was defined as a fractional inhibitory concentration index (FICI), as previously described [33].

$$\mathrm{FICI}=\frac{MIC\;of\;drug\;A\;in\;combination}{MIC\;of\;drug\;A\;alone}+\frac{MIC\;of\;drug\;B\;in\;combination}{MIC\;of\;drug\;B\;alone}$$

The FICI results for each combination were interpreted as follows: FICI ≤ 0.5, synergism; 0.5 < FICI < 1, additive; 1 ≤ FICI < 2, indifference; FICI ≥ 2, antagonism.

### 3.6. Time-Kill Assay

The bacteriostatic and bactericidal activity of different concentrations of antibiotics were investigated individually and in combination. Bacterial cultures (~10^6^ CFU/mL) were treated at MIC with imipenem, meropenem, doripenem, and rifampicin and in combinations of subinhibitory concentrations of rifampicin with either of the carbapenems over 24 h. Untreated cultures were maintained as controls. At intervals, (2, 4, 8, 12, and 24 h) aliquots of treated bacterial culture were analyzed by enumerating the bacterial count in CFU/mL. Results were presented as log reductions in CFU/mL, and all experiments were performed in triplicate for two independent repeats. Synergism was defined as a 2-log reduction in CFU/mL when compared with the most active single antibiotic treatment, whereas bactericidal activity was defined as a ≥3-log reduction in CFU/mL when compared with the number of viable cells at time zero (0 h) [34].

### 3.7. Biofilm Forming Assay

A crystal violet assay was conducted to identify biofilm-forming isolates among CRAB clinical isolates in 96-well microtiter plates [35], with slight modifications. Briefly, isolates were subcultured on TSA for 24 h, and a colony of each was inoculated into a sterile tube and grown overnight in MHB. The culture was adjusted to 10^6^ CFU/mL and resuspended in TSB. A 200 µL aliquot of each bacteria suspension was seeded in a 96-well microtiter plate and incubated for 24 h at 37 °C. Planktonic suspensions were aspirated, and the wells were washed twice with 300 µL of sterile phosphate-buffered saline (PBS). Plates were drained completely and allowed to dry in the laminar hood for 1 h. Completely dried wells were stained with 200 µL of 0.1% of crystal violet for 30 min. After staining, the excess crystal violet was aspirated, and the wells were washed with distilled water to remove residual crystal violet dye. The plates were again dried in the incubator until the wells were completely dried. The crystal violet absorbed in the biofilm biomass was solubilized in 200 µL of DMSO, and the absorbance was measured at an OD of 595 nm using the multimode plate reader EnSpire. Blank wells with media alone were maintained as negative controls, and all experiments were done in triplicates for three independent repeats. The isolates were classified into four groups based on the ability to form biofilm, following the interpretation criteria as below: OD_cut_ = OD_avg_ of negative control + SD of OD of negative control OD ≤ OD_cut_ = non-biofilm formers, OD_cut_ < OD ≤ 2 × OD_cut_ = weak biofilm formers, 2 × OD_cut_ < OD ≤ 4 × OD_cut_ = Moderate biofilm former, OD > 4 × OD_cut_ = Strong biofilm former [36].

### 3.8. Viability of Biofilm Cells

The MTT (3-(4, 5-dimethylthiazolyl-2)-2, 5-diphenyltetrazolium bromide) assay was employed to monitor the effects of single antibiotic treatment on the viability of biofilm cells [37]. Briefly, 1 mL of 10^6^ CFU/mL of bacterial suspension in TSB was seeded in a 24-well microtiter plate and incubated at 37 °C for 96 h. Fresh media were added at intervals to ensure that the wells were not dried. After the incubation period, unattached cells were aspirated, and plates were carefully washed with PBS without disruption of the sessile cells. Wells were treated with 16, 8, and 4 MICs of the respective antibiotics, and the plates were then incubated for 24 h at 37 °C. Untreated wells with bacteria alone were maintained as a control. Antibiotics were removed, and the plates were incubated with 300 µL of 0.05% MTT dye for 2 h. MTT-treated wells were then washed and dried before solubilizing with DMSO. The absorbance was taken at OD_595_, and all experiments were performed in triplicate for three independent repeats.

Adjunctive antibacterial therapy of rifampicin and carbapenems on 96 h-established biofilm was also investigated. The 96 h-established biofilms were treated with 500 µL of imipenem, meropenem, and doripenem in combination with 500 µL of rifampicin at various concentrations, including 8 MIC + 4 MIC, 8 MIC + 2 MIC, 4 MIC + 4 MIC, and 4 MIC + 2 MIC, respectively. Staining with MTT dye was done accordingly. Experiments were conducted in duplicate and repeated thrice. All results were presented as:

$$Percentage\;\left(\%\right)\;biofilm\;inhibition=\frac{Average\;mean\;of\;treated\;wells\;}{Average\;mean\;of\;untreated\;wells}\times 100$$

### 3.9. Scanning Electron Microscopy (SEM)

The effects of antibiotic treatments on bacteria cell membranes were investigated using a scanning electron microscope, as previously described, with modifications [38]. In brief, an overnight bacteria suspension was adjusted to 10^6^ CFU/mL and treated with several concentrations of antibiotic monotherapy at the MICs of the individual antibiotics and in combinations at MIC + 1/2 MIC, 1/2 MIC + 1/2 MIC, and 1/2 MIC+ 1/4 MIC of rifampicin and meropenem, respectively. Untreated bacterial cultures were used as a control. All tubes were further incubated at 37 °C for 3 h with constant agitation at 150 rpm. Cells were then harvested at 8000 rpm for 5 min and resuspended in PBS. Briefly, 100 µL of 10^8^ CFU/mL of washed bacterial cells was fixed on a glass slide using 3% glutaraldehyde solution for 2 h. Cells were dehydrated with concentration gradient ethanol (20, 40, 60, 80, and 100%) at an interval of 15 min. Samples were dried and gold coated before microscopy.

### 3.10. Statistical Analysis

Dunnett multiple comparison tests were used for the analysis of the data obtained at a significant level of the test and control at *p* < 0.05 with the Prism software.

## 4. Conclusions

The study demonstrated that the combination of rifampicin and carbapenem improved antibiofilm activity at lowered antibiotic concentrations. Carbapenem and rifampicin showed synergistic activity in inhibiting bacteria biofilm and bacteria membrane disruption. The combination reduced the survival rate within the biofilm environment better than the individual antibiotics in a dose-dependent manner. Although combination therapy did not significantly improve the antimicrobial efficacy, it significantly inhibited the biofilm viability, suggesting that the concurrent administration of antibiotics in combination may lead to the total eradication of the indwelling population of cells, making it easier to kill all unattached cells. Based on the results, we recommend that the efficacy of rifampicin combination with carbapenems should be further evaluated on young biofilm, biofilm eradication, and quorum sensing inhibition.

## Figures and Tables

**Table 1:** Minimum inhibitory concentration (MIC) and combinatorial antibacterial activity of subinhibitory concentration of carbapenems on carbapenem-resistant *Acinetobacter baumannii*.

Isolate Code	ZOI (mm)	MICs (µg/mL)		FICI	MICs (µg/mL)		FICI	MICs (µg/mL)		FICI
		RIF (RIF + MEM)	MEM (MEM + RIF)		RIF (RIF + DOR)	DOR (DOR + RIF)		RIF (RIF + IMP)	IMP (IMP + RIF)	
ST002	17.5	2 (0.5)	32 (16)	0.8 (A)	2 (0.5)	16 (8)	0.8 (A)	2 (1)	32 (16)	1(I)
ST004	NZ	64 (8)	16 (8)	0.6 (A)	64 (8)	16 (8)	0.6 (A)	64 (16)	128 (32)	0.6 (A)
ST011	7	1 (0.125)	128 (64)	0.6 (A)	1 (0.125)	32 (16)	0.6 (A)	1 (0.25)	512 (256)	0.8 (A)
ST016	5.8	1 (0.25)	16 (8)	0.8 (A)	ND	ND	ND	1 (0.5)	16 (8)	1 (I)
PA025	17.7	1 (0.5)	64 (32)	1 (I)	1 (0.5)	16 (0.5)	1 (I)	1 (0.13)	128 (64)	0.6 (A)
PA037	17.1	2 (0.5)	128 (32)	0.5 (S)	2 (0.5)	64 (32)	0.8 (A)	1 (0.5)	128 (32)	0.5 (S)
TR009	NZ	64 (16)	128 (64)	0.8 (A)	64 (16)	16 (8)	0.8 (A)	64 (16)	64 (16)	0.5 (S)
TR023	11.55	2 (0.5)	128 (32)	0.5 (S)	2 (05)	32 (16)	0.8 (A)	1 (0.25)	128 (32)	0.5 (S)
TR045	8	2 (0.5)	128 (64)	0.8 (A)	2 (0.5)	64 (32)	0.8 (A)	2 (0.5)	128 (64)	0.8 (A)
TR057	8.7	4 (1)	64 (16)	0.5	4 (1)	32 (16)	0.8 (A)	4 (1)	128 (64)	0.8 (A)
TR069	NZ	32 (4)	32 (16)	0.6 (A)	32 (4)	8 (4)	0.6 (A)	32 (4)	64 (32)	0.6 (A)
TR082	NZ	32 (4)	16 (8)	0.6 (A)	32 (4)	16 (8)	0.6 (A)	32 (4)	32 (16)	0.6 (A)
TR119	14.7	2 (0.5)	128 (64)	0.8 (A)	2 (0.5)	64 (32)	0.8 (A)	2 (0.5)	256 (128)	0.8 (A)
TR0121	12.7	2 (0.5)	128 (64)	0.8 (A)	2 (0.5)	64 (32)	0.8 (A)	2 (0.5)	256 (64)	0.5 (S)
TR123	7	8 (4)	128 (32)	0.8 (A)	8 (2)	128 (32)	0.5 (S)	8 (2)	64 (32)	0.5 (S)
TR125	NZ	64 (16)	16 (4)	0.5 (S)	64 (16)	8 (4)	0.8 (A)	64 (16)	16 (4)	0.5 (S)
TR131	10.2	4 (1)	32 (8)	0.5 (S)	4 (1)	8 (4)	0.8 (A)	4 (0.5)	32 (16)	0.6 (A)
SK009	10.69	256 (64)	128 (64)	0.8 (A)	256 (128)	64 (32)	1 (I)	256 (128)	64 (32)	1 (I)
SK015	NZ	64 (8)	64 (32)	0.6 (A)	64 (8)	32 (16)	0.8 (A)	64 (32)	64 (16)	0.6 (A)
SK024	NZ	16 (4)	128 (16)	0.5 (S)	16 (4)	16 (8)	0.8 (A)	16 (2)	64 (32)	0.6(A)
SK025	NZ	16 (2)	64 (32)	0.6 (A)	16 (4)	16 (8)	0.8 (A)	16 (2)	128 (64)	0.6(A)
SK035	7	1 (0.5)	32 (8)	0.8 (A)	1 (0.25)	32 (8)	0.5 (S)	1 (0.25)	64 (16)	0.5 (S)
SK040	NZ	32 (8)	16 (4)	0.5 (S)	32 (4)	16 (8)	0.6 (A)	32 (8)	64 (16)	0.5 (S)
SK052	NZ	64 (8)	32 (16)	0.6 (A)	64 (16)	32 (16)	0.8 (A)	64 (8)	128 (64)	0.6 (A)
SK056	NZ	64 (8)	32 (16)	0.6 (A)	64 (16)	16 (8)	0.8 (A)	64 (8)	64 (32)	0.6 (A)
SK059	NZ	64 (8)	16 (8)	0.6 (A)	64 (8)	8 (4)	0.6 (A)	64 (8)	32 (8)	0.4
Sk065	NZ	32 (4)	32 (16)	0.6 (A)	32 (4)	16 (8)	0.6 (A)	32 (4)	128 (32)	0.4 (S)
Sk067	14.7	64 (8)	64 (32)	0.6 (A)	64 (16)	32 (16)	0.8 (A)	64 (16)	256 (128)	0.8 (A)
SK068	14.7	1 (0.125)	128 (64)	0.6 (A)	1 (0.25)	32 (16)	0.8 (A)	1 (0.125)	128 (64)	0.6 (A)
ATCC 19606		<1	<1		<1	<1		<1	<1	

ZOI, zone of inhibition; RIF, rifampicin; MEM, meropenem; DOR, doripenem; IMP, imipenem; FICI, fractional inhibitory concentration index; ND, not determined; NZ, no zone; S, synergy: A, addictive; I, indifferent.

## Data Availability

Data is contained within the article and Supplementary Materials.

## Supplementary Materials

The following supporting information can be downloaded at: https://www.mdpi.com/article/10.3390/ph16040477/s1, Figure S1: The distribution of rifampicin-resistant *Acinetobacter baumannii* clinical isolates; Figure S2: Time-kill kinetic of rifampicin and combination with meropenem against rifampicin resistant and carbapenem resistant clinical isolate of *A. baumannii* SK024; Figure S3: Cell viability of monotherapy against 96 h established biofilm (A) TR069, (B) ST004, and (C) ATCC 19606 expressed as percentage viability; Figure S4: Cell viability of combination therapy against 96 h established biofilm (A) TR069, (B) ST004 (C), and ATCC 19606 expressed as percentage viability.
