# Supplementary material for: Rifampicin Enhanced Carbapenem Activity with Improved Antibacterial Effects and Eradicates Established Acinetobacter baumannii Biofilms
Source: Pharmaceuticals (Basel). 2023 Mar 23;16(4):477. doi: 10.3390/ph16040477 (PMC10141143; doi:10.3390/ph16040477)
Supplement: Supplementary file 1 [file pharmaceuticals-16-00477-s001.zip › pharmaceuticals-2270712-supplementary.pdf]

Rifampicin enhanced carbapenem activity with improved antibacterial effects and eradicates established *Acinetobacter baumannii* biofilms.

Lois Chinwe Nwabor[1], Arnon Chukamnerd[2], Ozioma Forstinus Nwabor[2], Rattanaruji Pomwised[3], Supayang P. Voravuthikunchai[3,4,5] and Sarunyou Chusri[1,2,] *

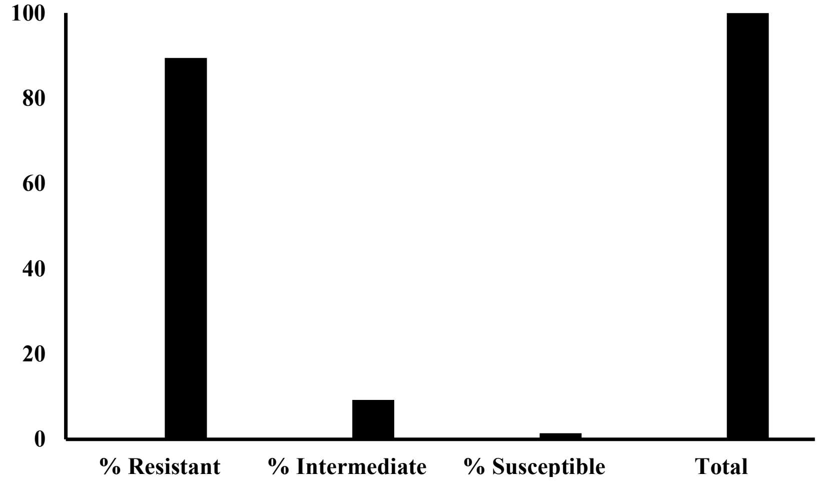

Figure S1: The distribution of rifampicin resistant *Acinetobacter baumannii* clinical isolates. CLSI interpretation criteria for *Staphylococcus aureus* * susceptible ≥ 20* intermediate 17-19* Resistant ≤ 16

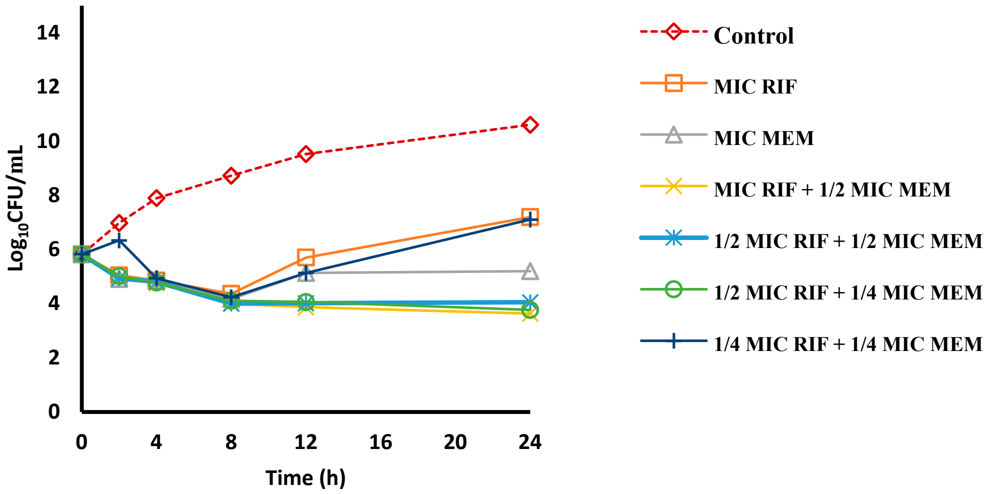

Figure S2. Time-kill kinetic of rifampicin and combination with meropenem against rifampicin resistant and carbapenem resistant clinical isolate of *A. baumannii* SK024 Experiments was performed in duplicate.

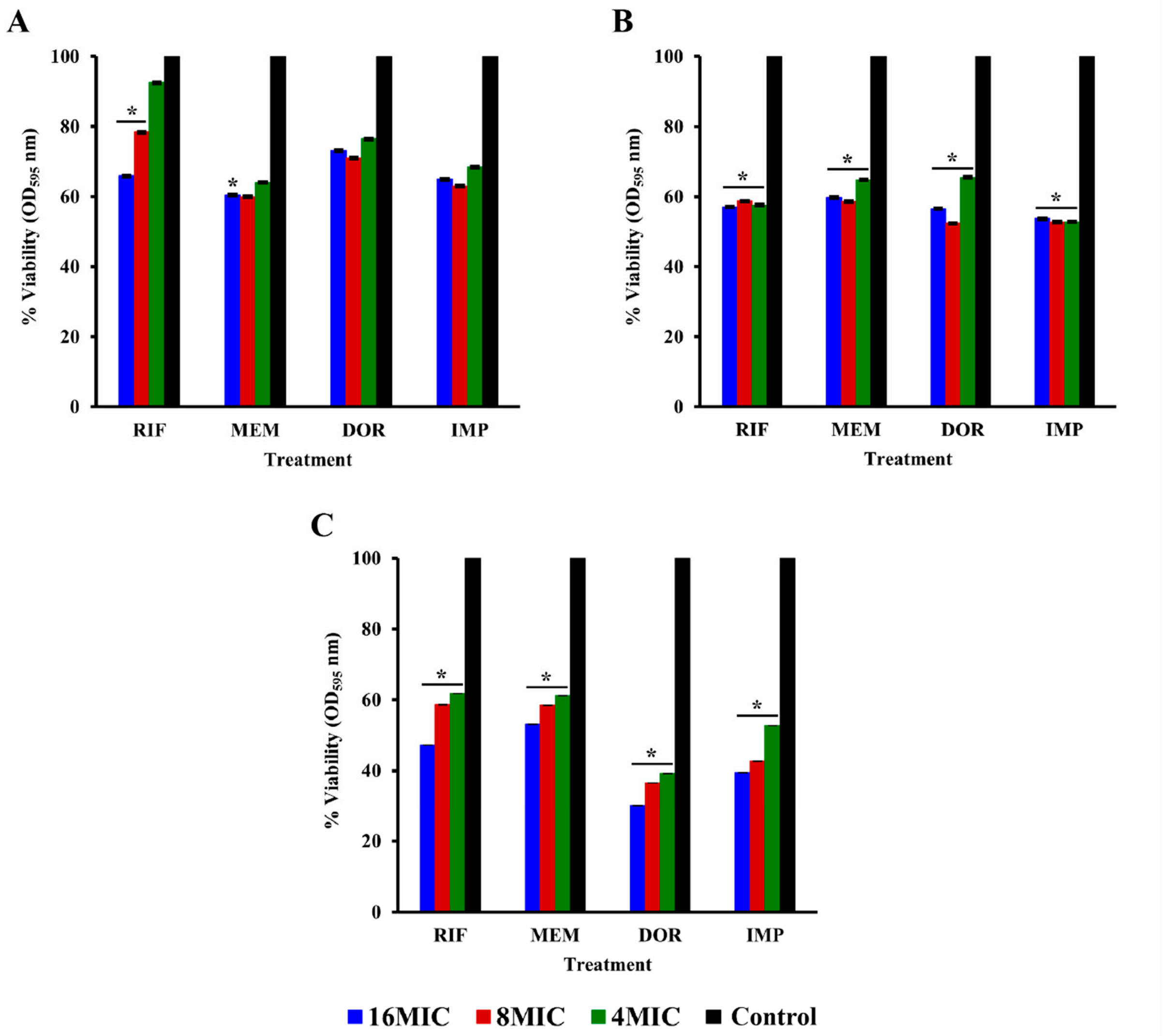

Figure S3. Cell viability of monotherapy against 96 h established biofilm (A) TR069 (B) ST004 and (C) ATCC 19606 expressed as percentage viability. MIC, minimum inhibitory concentration; RIF, rifampicin; MEM, meropenem; DOR, doripenem; IMP, imipenem. * $P < 0.05$

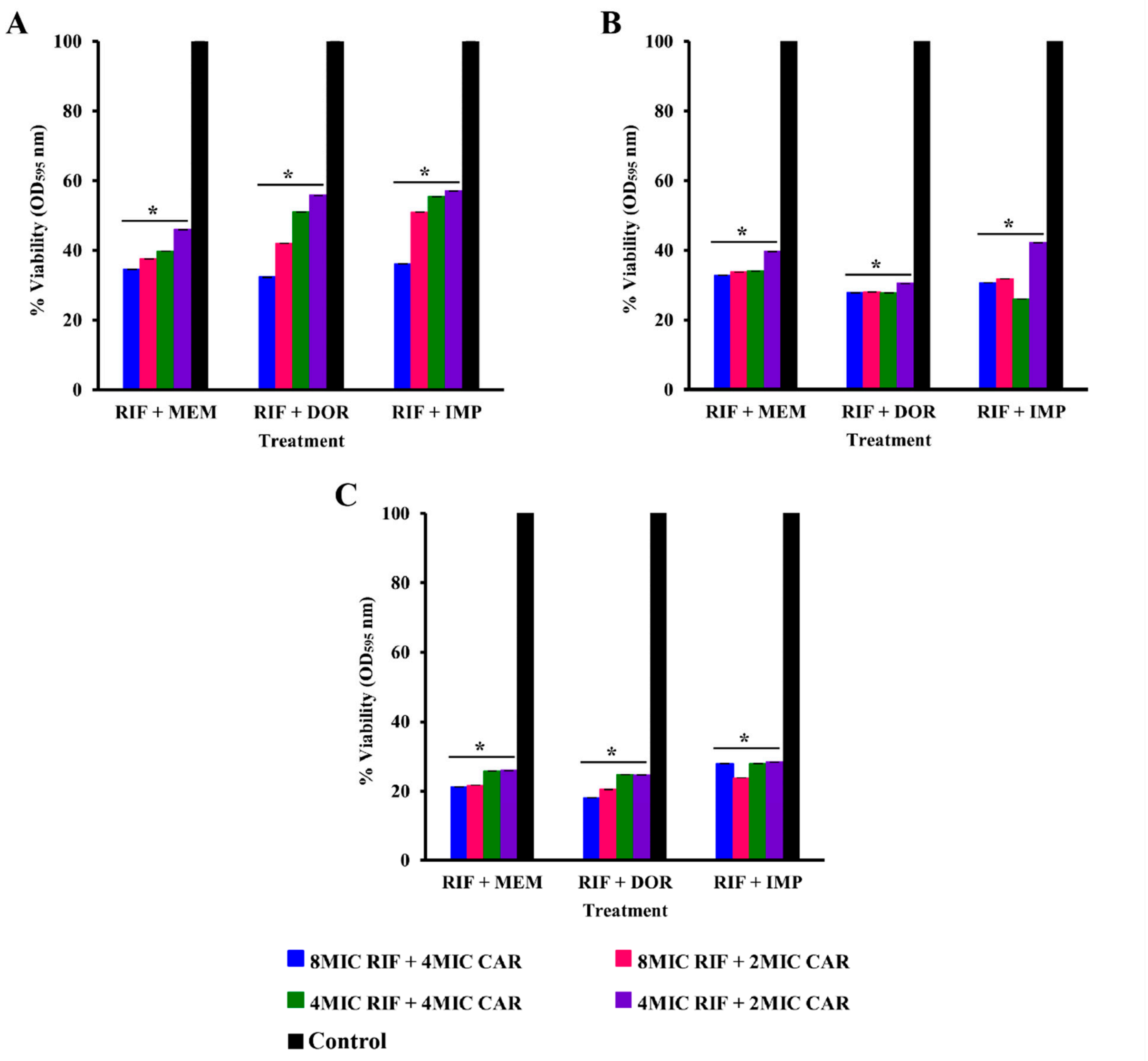

Figure S4. Cell viability of combination therapy against 96 h established biofilm (A) TR069 (B) ST004 and (C) ATCC 19606 expressed as percentage viability. MIC, minimum inhibitory concentration; RIF, rifampicin; MEM, meropenem; DOR, doripenem; IMP, imipenem. * $P < 0.05$
